# The continuous type of splenogonadal fusion: A rare case report and literature review

**DOI:** 10.1016/j.ijscr.2023.109006

**Published:** 2023-11-04

**Authors:** Quang Nguyen, Duy Khanh Nguyen, Huu Thao Nguyen, Xuan Truong Bui

**Affiliations:** a Center for Andrology and Sexual Medicine, Viet Duc University Hospital, Hanoi, Viet Nam; b University of Medicine and Pharmacy, Vietnam National University, Hanoi, Viet Nam

**Keywords:** Splenogonadal fusion, Inguinal hernia, Cryptorchidism, Laparoscopic, Case report

## Abstract

**Introduction:**

We report a rare case of the continuous type of splenogonadal fusion (SGF) in a young adolescent with preserved testis.

**Case presentation:**

A 19-year-old male patient with a history of left inguinal hernia repair 10 years ago presented with a palpable mass on the left side. Computed tomography revealed a 58x37mm mass with a tissue density of 47HU, demonstrating vigorous enhancement following contrast administration and displaying well-defined margins with the left testicle. It was noted to be growing vertically in the left inguinal canal and to be continuous with the lower pole of the native spleen. The patient underwent laparoscopic surgery to remove the splenic tail in the abdomen and to separate the scrotal spleen from the left testicle through the left inguinal tract. The histopathological examination confirmed the presence of splenic tissue.

**Discussion:**

SGF is often diagnosed incidentally during exploration or surgery for scrotal swelling or mass, cryptorchidism, or inguinal hernia in young patients. It is important to be aware of this condition to avoid unnecessary radical orchiectomy.

**Conclusion:**

Diagnosing the SGF preoperatively can be challenging. However, a combination of imaging modalities and negative tests for alpha-fetoprotein (AFP), lactate dehydrogenase (LDH), and beta-human chorionic gonadotropin (b-HCG) can aid in making an initial diagnosis. The use of laparoscopic surgery can further improve the diagnostic process, allowing clinicians to accurately diagnose SGF and make well-informed treatment decisions.

## Highlights

• Multi-scan computed tomography showed a left scrotal mass continuous to the lower pole of the native spleen which is typical of SGF.
• Laparoscopy revealed a typical appearance of the splenic tail, further strengthening the diagnosis of SGF.
• Case report of the continuous type of SGF, the first of its kind in Vietnam in the English literature.

## 1 Introduction

Splenogonadal fusion (SGF) is a rare benign congenital malformation characterized by an abnormal association between the splenic tissue and the gonads or mesonephric remnants [1,2]. SGF may be divided into continuous or discontinuous types. The continuous type of SGF occurs when the normally located spleen is attached to the gonad by a discrete cord. The discontinuous type consists of gonadal fusion with an accessory spleen or ectopic splenic tissue and completely separated from the normal spleen. SGF lacks typical clinical symptoms and is usually associated with other congenital malformations such as inguinal hernia and cryptorchidism, or it may only involve testicular masses. Due to insufficient understanding of the disease, it could be misdiagnosed as testicular malignancy and lead to unnecessary, life-altering orchiectomy [[3], [4], [5], [6]]. For educational and clinical purposes, we present a case report of a 19-year-old male with continuous type SGF, accompanied by an accessory spleen. The patient was successfully diagnosed preoperatively and underwent an accessory splenectomy and testicular preservation. This paperwork has been reported in line with the SCARE criteria and guidelines [7].

## 2 Case presentation

### 2.1 Patient information

A 19-year-old male patient, who is a university student and resides in Thai Binh province, Vietnam, has an average family income.

### 2.2 Clinical findings

The patient noticed a mass in the left scrotum for the past 3 months, which increased in size and did not cause any pain. Regarding his past medical history, he was a healthy-term baby with a previous history of a left inguinal hernia. He underwent open surgery at ten years old, during which this defect was not detected.

**Fig. 1:**
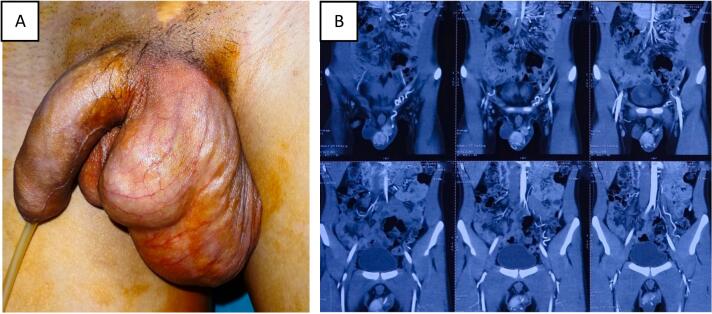
Clinical and subclinical signs. Fig. 1 A. The mass in the left scrotum is continuous with the testicle, and it pushes the testicle downward. Fig. 1 B. Multi-scan computed tomography showed a left scrotal mass consecutive to the lower pole of the native spleen due to SGF disease.

Clinical examination showed that the patient was in average physical condition with a BMI of 20. The external shape of the penis was normal, and the pubic hair was at Tanner stage 4. The left testicle can be palpated and the mass firmly attached right next to the left testicle was 4x3cm in size, firm, with clear boundaries, gradually tapering to the external inguinal ring (Fig. 1A).

### 2.3 Diagnostic assessment

First, during the ultrasound, we observed a mass located next to the left testicle. The mass had a volume of 20 cm3 and appeared to have a similar structure to testicular tissue. However, it was hypoechoic and mildly hypervascular. Based on these findings, my sonographer suspected a third testicle was present in the scrotum. Additionally, we conducted an abdominal ultrasound, chest X-ray, and echocardiography, which did not detect anything abnormal. An assay for testicular tumor markers was requested: AFP, b-HCG, and LDH were found to be normal. Multi-scan computed tomography detected a mass measuring 57x38mm, tissue density (47HU), strong enhancement after injection, mass with clear margins and boundaries pushing the testicles down, growing along the left inguinal canal up to left iliac fossa and continuous with the lower pole of the spleen (Fig. 1B). The preliminary diagnosis given was SGF.

### 2.4 Therapeutic methods

**Fig. 2:**
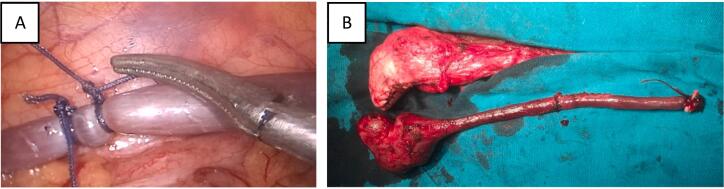
The surgery involves the removal of the splenic tail (Fig. 2A) and the separation of the scrotal spleen (Fig. 2B).

The patient underwent exploratory laparoscopic surgery and it was observed that the malformed spleen grew from the lower pole of the spleen. It followed the colonic groove and entered the inguinal canal, ultimately sticking to the testicles. At this time, the diagnosis of SGF was confirmed. We performed a resection of the splenic tail in the abdomen (Fig. 2A) and surgically separated the scrotal spleen from the testicle while preserving the left testicle through the left inguinal line (Fig. 2B).

The histopathological examination confirmed the presence of splenic tissue.

### 2.5 Outcomes

After surgery, the patient was stable, had no short-term complications such as bleeding or infection, and was discharged after 3 days. We schedule patient follow-up visits after 1 month and 6 months to monitor complications such as testicular atrophy, and recurrence, and evaluate the patient's fertility.

## 3 Discussion

SGF is a very rare congenital anomaly and was first documented by Bostroem in 1883 [1]. A comprehensive analysis of 123 cases was conducted by Carragher in 1990 [2], and an additional 61 SGF cases were published by Malik et al. in 2013 [8]. Since then, Guangjie Chen et al. have analyzed 41 additional cases written in English up to 2020 [5]. We utilized data from PubMed and identified 21 newly reported cases of open-access surgery, including our cases summarized in Table 1 [4,5,[9], [10], [11], [12], [13], [14], [15], [16], [17], [18], [19], [20], [21], [22], [23], [24], [25], [26]]. Additionally, there were 4 cases of non-open-access surgery [[27], [28], [29], [30]]. Therefore, as of 2023, a total of approximately 250 SGF cases have been reported.

**Table 1:** Summary of characteristics of SGF cases (*N* = 21).

		Number (N = 21)
Age at diagnosis (years), n (%)	0–9	11 (52,4 %)
	10–19	4 (19 %)
	20–30	3 (14,3 %)
	>30	3 (14,3 %)
Gender, n (%)	Male	20 (95,2 %)
	Phenotypical female (phenotypical sex reversal)	1 (4,8 %)
Side, n (%)	Left	21 (100 %)
	Right	0 (0 %)
Classification, n (%)	Continuous	10 (48 %)
	Discontinuous	7 (33 %)
	Unknown	4 (19 %)
Clinical presentation, n (%)	Testicular swelling/mass	14 (66,7 %)
	Cryptorchidism	7 (33,3 %)
Congenital anomalies associated with SGF, n (%)	Cryptorchidism	11 (52,4 %)
	Inguinal hernia	5 (23,8 %)
	Hypospadias	1 (4,7 %)
	ventricular septal defect, patent ductus arteriosus, and coarctation of aorta	1 (4,7 %)
	Cleft palate, right pectoralis major hypoplasia, disruptive defect of the right upper limb	1 (4,7 %)
	Phenotypical sex reversal	1 (4,7 %)
	Hepatogonadal fusion	1 (4,7 %)
	None	4 (19 %)
Testicle preservation, n (%)	Orchiectomy	10 (48 %)
	Preservation	11 (52 %)

This anomaly concerns almost exclusively the male sex, with a male-to-female ratio of 14–15:1 [8,31]. However, there have been two reported cases of female individuals with a genotype of 46, XY (phenotypical sex reversal) [8,13,31,32]. In men, this condition is more common due to the male gonads being located outside the body, while in women it tends to cause fewer symptoms and complications. SGF is frequently observed in children and adolescents, with 70 % of cases occurring in individuals under the age of 20 [3]. In our reported cases, 15 out of 21 (71 %) were individuals under the age of 20 (Table 1).

The etiology of SGF remains incompletely understood; however, the prevailing hypothesis posits its occurrence during gestational weeks 5 to 8, before descent of the gonads commences. Between the 5th and 6th weeks of gestation, the spleen develops from the splenic anlage in the left dorsal mesogastrium, and the gonadal ridge is formed between the mesonephros and dorsal mesentery. During the 6th and 7th weeks of gestation, the dorsal mesogastrium rotates to the left, placing the splenic anlage into proximity with the left urogenital fold which contains the gonadal mesoderm. Such proximity remains until the descent of the gonads during the 8th week of gestation [1,2,8]. The proximity between the left gonad and spleen during embryogenesis explains the fact that SGF almost always occurs on the left side. This adhesion may disrupt the normal formation and migration process of the gonads, leading to associated anomalies such as cryptorchidism and inguinal hernia in the majority of SGF cases [2,5,8,33].

SGF can be classified into 2 types, based on its relationship with the native spleen. Continuous SGF is characterized by the presence of a cord of splenic or fibrous tissue connecting the spleen and gonad, and occasionally beads of splenic tissue could be found along the cord (splenic rosary bead), while in discontinuous SGF, there is no such connection. Rather, in discontinuous SGF, ectopic splenic tissue or accessory spleen is directly attached to the gonad without connecting to the native spleen [2,5,34,35]. 10 (48 %) of the 21 cases in our reported cases were continuous, and 7 (33 %) cases were discontinuous and 4 cases were unclassified because the author only discovered the relation through histopathology after orchiectomy and was unsure if it was related to the native spleen (Table 1). During embryonic development, the descent of the testis could sometimes draw out the developing spleen fused to the testis into a long band (Continuous SGF) or carry a portion of the splenic primordium down with the descending testis (Discontinuous SGF) [5]. The most common malformations associated with SGF are cryptorchidism and inguinal hernia, Malik et al. found that 36 % of SGF patients had cryptorchidism [8], with Chen G. et al. found that 29,27 % had cryptorchidism and 12,2 % had inguinal hernia [5]. In our summary, 16 of the 21 SGF cases (76 %) were associated with cryptorchidism (11 [52,4 %]) and inguinal hernia (5 [23,8 %]), among them, there were 2 cases with both malformations (Table 1).

SGF is often an incidental finding during exploration/surgery for scrotal swelling/mass, cryptorchidism, or inguinal hernia [5]. In our study, the clinical presentations were testicular swelling or mass (66.7 %) and cryptorchidism (33.3 %) (Table 1). The diagnosis of SGF is often overlooked due to its rarity. However, imaging techniques like B-mode ultrasound, computed tomography (CT), and MRI can be valuable in observing the typical imaging features of the continuous fibrous cord that connects the spleen and the testis in cases of continuous SGF. However, this can be challenging because SGF exhibits morphological diversity. Radiocolloid spleen scintigraphy (99mTc‑sulfur colloid spleen-liver scan) in CT scan can be useful to identify ectopic splenic tissue and thus could help to diagnose SGF when a surgeon has a high pre-operation suspicion of SGF. In our practice, ultrasound is used for preliminary screening for congenital anomalies. However, ultrasonographic imaging is not as beneficial as expected due to overlapping features of SGF and testicular malignancies and the diagnostic accuracy of ultrasound is affected by the experience of the examiners [16]. Meanwhile, CT and MRI are reliable and accurate in detecting the position and shape of the testis and in ruling out other congenital malformations [36]. On this basis, Youssef et al. proposed a diagnosis and treatment decision tree for SGF: ultrasound and tumor marker testing should be employed first. If tumor markers are elevated, orchiectomy can be performed directly; if tumor markers are normal but a testicular neoplasm is highly suspected by ultrasound, surgical exploration and intraoperative biopsy will be feasible for diagnosis [4,22]. In any case, diagnostic laparoscopy is recommended as it is safe, reliable, and very accurate in diagnosing and treating an impalpable testis [5,8].

We all know that SGF is a congenital disease, meaning that the benign tissue has existed and slowly developed along with the testis. Because it is a benign disease, preserving the testicle is always a top priority in treatment. However, according to a 1990 report, >37 % of SGF patients underwent an unnecessary orchiectomy [5]. In our report, the orchiectomy rate was 48 % (Table 1).

## 4 Conclusion

To the best of our knowledge, we present a case report of the continuous type of SGF, the first of its kind in Vietnam in the English literature. SGF is a rare benign birth defect that should be taken into account before performing orchiectomy, particularly when concurrent with cryptorchidism or inguinal hernia.

## Ethical approval

Not applicable as it is a case report.

## Funding

This report involved no sources of funding for any of the authors.

## CRediT authorship contribution statement

QN were the main doctors involved in conceiving the original idea; QN, DN, HN designed the study and gathered data. XB conceived the manuscript, edited it, performed the operation, and wrote the manuscript. All authors read and approved the final manuscript.

## Guarantor

Quang Nguyen.

## Informed consent

Written consent was obtained from the patient and the patient's father to publish this case report. A copy of the written consent is available for review by the Editor-in-Chief of this journal.

## Declaration of competing interest

The authors declare that the research was conducted in the absence of any commercial or financial relationships that could be construed as a potential conflict of interest.

## Data Availability

The original contributions presented in the study are included in the article/supplementary material. Further inquiries can be directed to the corresponding author.
